# ImmunoChip Study Implicates Antigen Presentation to T Cells in Narcolepsy

**DOI:** 10.1371/journal.pgen.1003270

**Published:** 2013-02-14

**Authors:** Juliette Faraco, Ling Lin, Birgitte Rahbek Kornum, Eimear E. Kenny, Gosia Trynka, Mali Einen, Tom J. Rico, Peter Lichtner, Yves Dauvilliers, Isabelle Arnulf, Michel Lecendreux, Sirous Javidi, Peter Geisler, Geert Mayer, Fabio Pizza, Francesca Poli, Giuseppe Plazzi, Sebastiaan Overeem, Gert Jan Lammers, David Kemlink, Karel Sonka, Sona Nevsimalova, Guy Rouleau, Alex Desautels, Jacques Montplaisir, Birgit Frauscher, Laura Ehrmann, Birgit Högl, Poul Jennum, Patrice Bourgin, Rosa Peraita-Adrados, Alex Iranzo, Claudio Bassetti, Wei-Min Chen, Patrick Concannon, Susan D. Thompson, Vincent Damotte, Bertrand Fontaine, Maxime Breban, Christian Gieger, Norman Klopp, Panos Deloukas, Cisca Wijmenga, Joachim Hallmayer, Suna Onengut-Gumuscu, Stephen S. Rich, Juliane Winkelmann, Emmanuel Mignot

**Affiliations:** 1 Center for Sleep Sciences and Medicine, Stanford University, Palo Alto, California, United States of America; 2 Center for Sleep Medicine, Department of Clinical Neurophysiology, Faculty of Health Sciences, University of Copenhagen, Glostrup Hospital, Copenhagen, Denmark; 3 Department of Genetics, Stanford University, Palo Alto, California, United States of America; 4 University of Groningen, University Medical Center Groningen, Department of Genetics, Groningen, The Netherlands; 5 Institute of Human Genetics, Helmholtz Zentrum München–German Research Center for Environmental Health, Munich, Germany; 6 National Reference Network for Orphan Diseases (Narcolepsy and Idiopathic Hypersomnia), Paris, France; 7 Sleep Unit, Gui-de-Chauliac Hospital, INSERM-1061, Montpellier, France; 8 Sleep Disorders Unit, Hospital Pitié-Salpêtrière, Pierre and Marie Curie University, Paris, France; 9 Centre Pédiatrique des Pathologies du Sommeil, Hôpital Robert Debré, Paris, France; 10 Hephata-Klinik, Schwalmstadt-Treysa, Germany; 11 Department of Neurology, Philipps University of Marburg, Marburg, Germany; 12 Department of Psychiatry and Psychotherapy, University of Regensburg, Regensburg, Germany; 13 Department of Neurological Sciences, University of Bologna/IRCCS Istituto delle Scienze Neurologiche, Bologna, Italy; 14 Kempenhaeghe Centre for Sleep Medicine, Heeze, The Netherlands; 15 Leiden University Medical Center, Department of Neurology, Leiden University Medical Center, Leiden, The Netherlands; 16 Department of Neurology, Charles University, 1st Faculty of Medicine and General Teaching Hospital, Prague, Czech Republic; 17 CHU Ste-Justine Research Centre, Centre of Excellence in Neuromics, Université de Montréal (CENUM), Montreal, Quebec, Canada; 18 Neurology Service, Hôpital du Sacré-Coeur, Université de Montréal, Montréal, Quebec, Canada; 19 Center for Advanced Research in Sleep Medicine, Hôpital du Sacré-Cœur, Université de Montréal, Montréal, Québec, Canada; 20 Department of Neurology, Innsbruck Medical University, Innsbruck, Austria; 21 University Sleep Clinic and CNRS UPR3212, Strasbourg University Hospital, Strasbourg University, Strasbourg, France; 22 Sleep and Epilepsy Unit-Clinical Neurophysiology Service, University Hospital Gregorio Marañón, Madrid, Spain; 23 Neurology Service and Multisciplinary Sleep Unit, Hospital Clínic, CIBERNED, IDIBAPS, Barcelona, Spain; 24 Department of Neurology, Inselspital Universitatsspital, Bern, Swizerland; 25 Center for Public Health Genomics, University of Virginia, Charlottesville, Virginia, United States of America; 26 Division of Rheumatology, Cincinnati Children's Hospital Medical Center, Cincinnati, Ohio, United States of America; 27 Inserm, U975, CRICM, Paris, France; 28 Pierre Marie Curie University, UMR-S975, Paris, France; 29 Assistance Publique-Hôpitaux de Paris, Department of Neurology, Hospital Pitié-Salpêtrière, Paris, France; 30 Cochin Institute, INSERM U1016/CNRS UMR 8104/Paris Descartes University, Paris, France; 31 Department of Rheumatology, Ambroise Paré Hospital AP-HP, Boulogne-Billancourt, France; 32 Université Versailles Saint Quentin en Yvelines (UVSQ), Boulogne-Billancourt, France; 33 Institute of Genetic Epidemiology, Helmholtz Zentrum München, Munich, Germany; 34 Wellcome Trust Sanger Institute, Hinxton, United Kingdom; 35 Department of Psychiatry, Stanford University School of Medicine, Palo Alto, California, United States of America; 36 Institute for Human Genetics, Klinikum rechts der Isar, Technische Universität München, Munich, Germany; 37 Neurology Clinic, Klinikum rechts der Isar, Technische Universität München, Munich, Germany; 38 Munich Cluster for Systems Neurology (SyNergy), Munich, Germany; The Jackson Laboratory, United States of America

## Abstract

Recent advances in the identification of susceptibility genes and environmental exposures provide broad support for a post-infectious autoimmune basis for narcolepsy/hypocretin (orexin) deficiency. We genotyped loci associated with other autoimmune and inflammatory diseases in 1,886 individuals with hypocretin-deficient narcolepsy and 10,421 controls, all of European ancestry, using a custom genotyping array (ImmunoChip). Three loci located outside the Human Leukocyte Antigen (HLA) region on chromosome 6 were significantly associated with disease risk. In addition to a strong signal in the T cell receptor alpha (TRA@), variants in two additional narcolepsy loci, Cathepsin H (*CTSH*) and Tumor necrosis factor (ligand) superfamily member 4 (*TNFSF4*, also called *OX40L*), attained genome-wide significance. These findings underline the importance of antigen presentation by HLA Class II to T cells in the pathophysiology of this autoimmune disease.

## Author Summary

While there is now broad consensus that narcolepsy-hypocretin deficiency results from a highly specific autoimmune attack on hypocretin cells, little is understood regarding the initiation and progression of the underlying autoimmune process. We have taken advantage of a unique high-density genotyping platform (the ImmunoChip) designed to study variants in genes known to be important to autoimmune and inflammatory diseases. Our study of nearly 2000 narcolepsy cases compared to 10,000 controls underscored important roles for HLA DQB1*06:02 and the T cell receptor alpha genes and implicated two additional genes, Cathepsin H and *TNFSF4/OX40L*, in disease pathogenesis. These findings are particularly important, as these encoded proteins have key roles in antigen processing, presentation, and T cell response, and they suggest that specific interactions at the immunological synapse constitute the pathway to the disease. Further studies of these genes and encoded proteins may therefore reveal the mechanism leading to this highly selective and unique autoimmune disease.

## Introduction

Narcolepsy is a life-long sleep disorder caused by the autoimmune-mediated loss of 70,000–90,000 hypocretin (orexin)-producing neurons in the hypothalamus. Prevalence is approximately 0.02–0.03% in Caucasian populations, and somewhat higher in Japanese (0.16%). Family and twin studies support the importance of genetic (10–40 fold increased risk in first degree relatives) as well as environmental factors (25% concordance in identical twins) [1]. Onset is typically around puberty and displays a seasonal pattern of incidence, with highest rates in spring and summer. Likely triggering factors are influenza A, notably the pandemic H1N1 2009 variant, and *Streptococcus Pyogenes* infections [2]–[5]. Unique among autoimmune diseases, the condition is almost completely associated with Human HLA DQ0602, a heterodimeric protein encoded by the DQA1*01:02-DQB1*06:02 haplotype (90% versus 25% frequency in European ancestry cases and controls, respectively). The overwhelming effect of this haplotype on risk suggests the importance of antigen presentation by DQ0602. As seen in other autoimmune diseases, additional HLA alleles carried in trans of this haplotype also confer modulatory effects [6], [7]. Most notably, DQA1*01:02-DQB1*06:02 homozygosity increases predisposition by 2–4 fold. Further, DQA1 and DQB1 alleles known to heterodimerize with DQA1*01:02 or DQB1*06:02 reduce susceptibility, likely through allelic competition with DQ0602 [8]. However, non-HLA related genes play important roles. In addition to these well-established HLA class II effects, recent genome-wide association studies (GWAS) have identified variants in the T Cell receptor alpha locus (TRA@), on chromosome 14q11.2, and in the region containing *P2RY11-DNMT1* on chromosome 19p13.2, as additional susceptibility loci. Finally, exome sequencing in families with a rare autosomal dominant syndrome including cerebellar ataxia, narcolepsy and deafness (ADCA-DN) also indicate an important role for DNMT1 in survival of hypocretin neurons [9].

**Figure 1:**
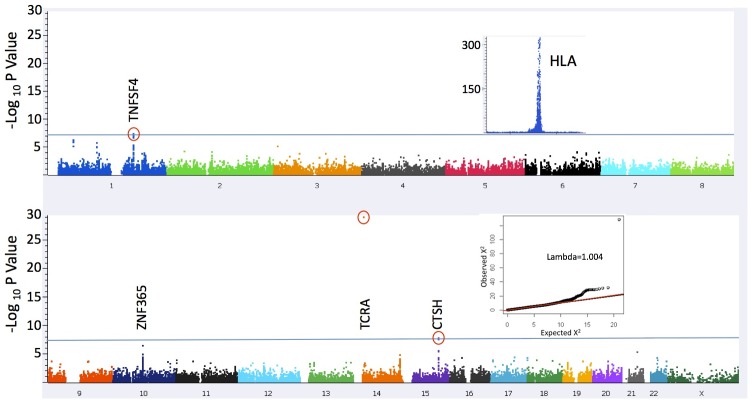
Manhattan Plot of association statistics. The significance threshold used (blue line) was P = 5×10^−8^; The insets depict plots of 1) association results in a broad region encompassing the HLA locus (chr 6:24,067–35,474 kb) that were excluded from the present analysis (see Methods) and 2) QQ plot of results for 109,777 markers after excluding a 1 Mb window surrounding the associated loci (λ = 1.004). The inflation statistic for all 111,240 tested markers is 1.04.

Based on the recognition that considerable overlap exists in risk loci for various autoimmune diseases, the Immunochip consortium was formed to create a single nucleotide polymorphism (SNP) array for targeted finemapping of these loci [10], [11]. The ImmunoChip was designed for deep replication of signals from large-scale meta-analyses in nine autoimmune diseases, and for finemapping of loci reaching genome-wide significance (2009). Approximately 200,000 rare and common variants were selected to cover intervals with established genome-wide significant association to autoimmune and seronegative diseases, and at selected loci of known importance in major immune-related diseases, including the major histocompatibility (MHC) and *KIR/LILR* loci. Using this platform, we conducted a GWAS to identify genetic risk factors for narcolepsy in addition to HLA DQ0602. We analyzed 111,240 high quality SNP markers of minor allele frequency ≥1%, located outside the extended HLA region on chromosome 6, in 1,886 narcolepsy cases and 10,421 controls of European ancestry sampled across global populations including the European Union, Canada and North America (Table 1). To test for potential confounding effects of population stratification in our study cohort, we performed principal component analysis (PCA) of cases and controls (Figure S1). Control samples showed clear separation into distinct European countries in plots of the first and second principal components, with good overlay of case samples, and plots of observed versus expected association results showed no inflation of signal (λ = 1.004; Figure 1).

**Table 1:** Sample collections.

Case Cohort	Number	Region of Origin
Virginia	1,030	North America, Europe*
Germany	801	North America, Europe*
Stanford VA	55	North America, Europe*
Total	1,886	
**Control Cohort**		
1958 UK Birth Cohort	4,289	UK
IT.NL.PL.SP	3,609	Italy, Netherlands, Poland, Spain
KORA	980	Germany
CCHMC	794	North America
Fr1	347	France
Fr2	402	France
Total	10,421	

* Numbers of samples by country of origin are listed in the Methods section.

Case cohort names represent location of genotyping, and do not reflect country of origin of samples.

## Results/Discussion

One previously reported, and two novel non-HLA loci surpassed genome wide significance (gws) P<5×10^−8^ in this study (Figure 1 and Table 2). The strongest association was with rs1154155 (MAF = 0.15, P = 8.87×10^−30^ OR = 1.72) in the T cell receptor (TCR) alpha (TRA@) locus, on chromosome 14, replicating signal previously reported using smaller samples [7], [12], [13]. The TCR protein is comprised of alpha and beta chains. As for immunoglobulin loci, TCR loci undergo somatic DNA recombination during T cell development, generating a large number of possible proteins specific to individual T-cell clones. T cells bearing specific recombinants are then negatively or positively selected, allowing adaptation of the immune system to past environmental history.

**Table 2:** Non-HLA narcolepsy risk variant loci reaching genome-wide significance.

Variant	Chr	BP	MAF_N	MAF_C	P	OR	CI	Locus	Risk allele
rs1154155	14	22072524	0.2292	0.1478	8.87×10^−30^	1.715	1.543–1.905	TCRA	G
rs34593439	15	77022012	0.1359	0.1053	1.78×10^−08^	1.337	1.212–1.455	CTSH	A
rs7553711	1	171398531	0.3462	0.2851	4.08×10^−08^	1.328	1.176–1.519	TNFSF4	C

Chr.: Chromosome; BP: position according to NCBI build 36 (Hg18) coordinates; MAF_N: minor allele frequency in narcolepsy (_N) and controls (_C); P: P value according to variance component model (EMMAX). EMMAX does not provide OR (Odds Ratio) or adjusted allele frequencies, therefore MAF, OR, and 95% confidence intervals (CI) were calculated with Plink on subset of 8,474 samples with the greatest PCA homogeneity (see Figure S2; EV 11.21<0.004, EV 4.12<0.01).

The T cell receptor binds foreign or self-peptides presented by Class II MHC proteins (such as the DQ alpha/beta heterodimer), allowing initiation and regulation of immune responses. It is thus the natural receptor of DQB0602. The TRA@ locus was sparsely covered on the ImmunoChip (15 SNPs within a 1 Mb window of rs1154155, none with r2 above 0.5) precluding fine mapping or haplotype analysis, although providing robust replication of the previously reported findings. SNP rs1154155 is located close to the J10 segment region of the locus, with linkage disequilibrium (LD) data suggesting the involvement of a specific J segment in the narcolepsy pathophysiology. The association with TRA@ is unique to narcolepsy, as no other autoimmune diseases have been associated with this locus.

**Figure 2:**
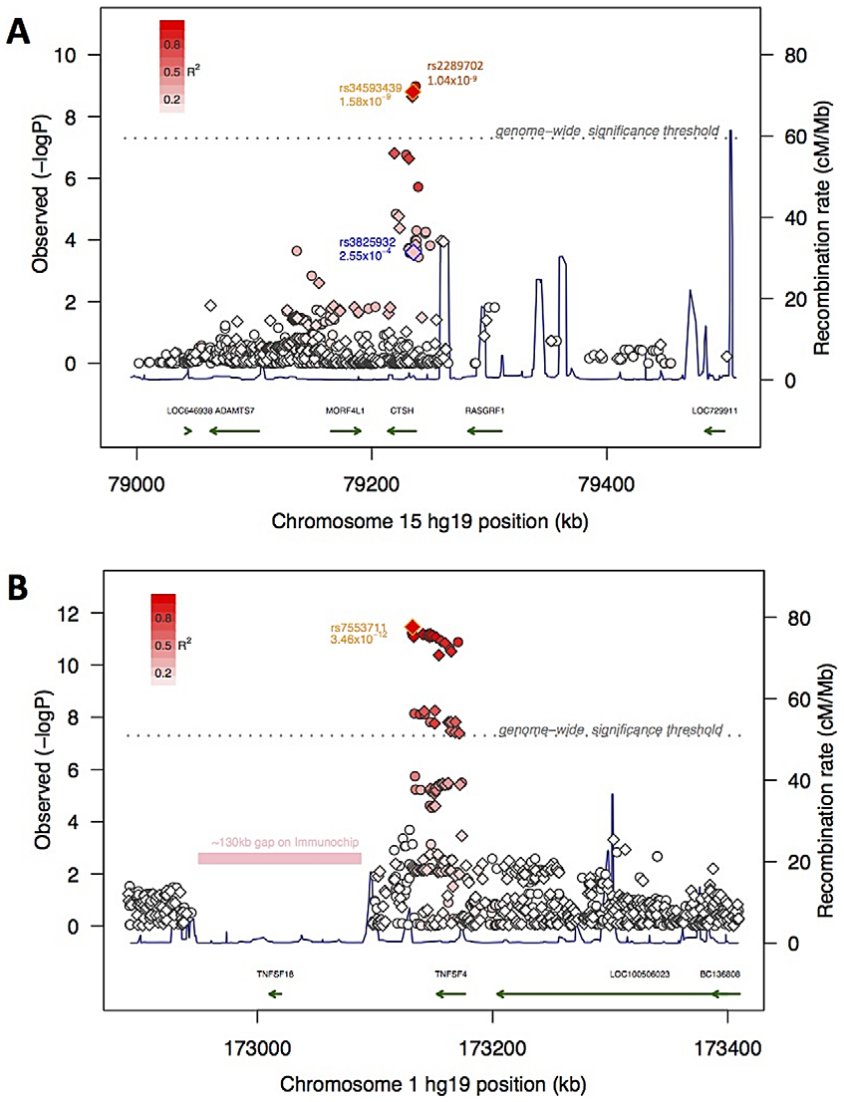
Association signal at the mapping intervals flanking rs34593439 and rs7553711. Association scores at 15q25.1 (panel A) and 1q25.1 (panel B). Genotyped (diamonds) and imputed (circles) SNPs are indicated and the top genotyped SNP in the interval is outlined in orange. A SNP in 15q25.1 previously associated with Diabetes is outlined in blue. The degree of red color in each diamond or circle indicates the strength of LD with the top SNP (on a scale shown in the legend at the upper left hand corner of the plot). The X-axis shows the chromosome and physical distance (kb) from the human genome reference sequence (hg19), the left Y-axis shows the negative base ten logarithm of the p-value and the right Y-axis shows recombination rate (cM/Mb) as a navy line. The genome-wide significance threshold (P<5×10^−8^) is given by the dashed grey line. Genes in the regions are annotated at the bottom as green arrows. Also indicated in 1q25.1 is a ∼130 kb region with no SNPs on the ImmunoChip.

Two SNPs rs34593439, and rs34843303, located in intron 1 of Cathepsin H (*CTSH*), a papain-like cysteine protease, reached gws (MAF = 0.11, P = 1.78×10^−8^ OR = 1.34 and MAF = 0.11, P< = 2.79×10^−8^ OR = 1.35, respectively). Another SNP located in intron 1, rs3825932T has been previously reported to be associated with type 1 diabetes [14], [15]. Although in close proximity, this marker is in weak LD with rs34593439 and rs34843303 (r2 = 0.23 and 0.23 respectively) and shows no significant association in the present sample (P = 0.01). The local region of LD surrounding these markers encompasses exon 1, where 4 potentially functional polymorphisms have been identified. One of which, SNP rs2289702T (p.Gly11Arg, MAF = 0.11), is in tight LD with our markers (pairwise r2 = 0.96 and 0.98 respectively, 1000genomes data, phase 1 release V3), and could be the culprit behind this association. Following imputation in a 1 Mb window surrounding *CTSH*, SNPs rs2289702 and rs34593439 were the two most highly associated variants (respectively) (Figure 2). The Arg allele of rs2289702 also underlies a minor histocompatability antigen restricted by HLA-A*3101 and HLA-A*3303, causing selective lysis of hematopoetic cells by cytotoxic lymphocytes [16]. p.Gly11Arg is located within the signal peptide sequence of CTSH, where the introduction of a highly charged arginine could affect trafficking or cleavage, as predicted by some but not all signal peptide predicting programs (see Methods).

Cathepsins are primarily located within the lysosomal/endosomal compartment and typically activated by low pH. These enzymes play diverse and important roles including cellular recycling of proteins, activation of selected preprohormones, antigen processing, and loading of peptides onto MHC class II proteins. Eleven family members are known. Cystatins and other endogenous inhibitors are known to regulate cathepsin activity, and the balance of these activities has been proposed to be the major selector for the repertoire of surface peptide –MHC II complexes [17]. Deficiencies in selected cathepsins impair immune cell development (NKT cells in Cathepsin S or L deficient mice, thymocytes and T cell repertoire in cathepsin L deficient mice), and produce defects in immune cell effector functions (cytotoxic T cell, neutrophil and mast cell defects in cathepsin C deficient mice; see [18]). Cathepsin H is somewhat unique in that it can have both exopeptidase and endopeptidase activities, depending on the presence of a bound mini-chain (a remnant of the pro-enzyme) within the active cleft. Although ubiquitously expressed, *CTSH* expression is especially high in type II pneumocytes, where it plays a key role in the maturation of lung surfactant protein B [17], [19]. *CTSH* is also highly expressed in MHC class II positive immune cells such as B cells, monocytes and dendritic cells, but not T cells, notably in the presence of inflammation. For example, CTSH enzyme activity increases in parallel with proinflammatory cytokines during the development of autoimmune inflammation in a NOD mouse model of Sjögren's syndrome [20]. One hypothesis may be that decreased CTSH activity reduces antigen processing resulting in an altered repertoire presented by DQB0602 and resulting in increased risk of narcolepsy.

SNPs located in the Tumor Necrosis Factor (ligand) Superfamily member 4 (*TNFSF4;* also called *OX40L* or *CD252*) are strongly associated with narcolepsy. SNP rs7553711 reached gws (MAF 0.29, P = 4.08×10^−8^ and OR = 1.33). No other SNP was more strongly associated with narcolepsy following imputation in a 1 Mb window around this locus, although additional strongly associated variants were identified (Figure 2). *TNFSF4* is known to be strongly associated with systemic lupus erythematosis (SLE) [21] and systemic sclerosis [22], [23] and SNPs in this region were densely represented on the ImmunoChip. Two distinct haplotypes composed of SNPs upstream of the gene confer susceptibility or resistance to SLE, whereas our most significantly associated SNP markers in narcolepsy are downstream of the gene in a separate haplotype block. The SNPs associated with SLE and narcolepsy are in weak LD, and rs844648, an established marker of SLE, is not strongly associated with narcolepsy (p = 0.016). Interestingly, rs7553711 maps to a potential enhancer site (H3K4Me1 site, UCSC browser, Layered H3K27 Track).

The association of narcolepsy with SNPs in *TNFSF4* is consistent with a primary role of antigen presentation to T cells in narcolepsy. Like *CTSH*, *OX40L* is primarily expressed in MHC Class II-positive antigen presenting cells (*e.g.* dendritic and B cells). Optimal activation of T cells following the binding of T cell receptor- MHC class II/antigen complex requires the action of additional costimulatory factors, notably involving receptor/ligand pairs from the tumor necrosis superfamily. The interaction of two of these, OX40 receptor (encoded by *TNFRSF4*) and OX40L ligand (encoded by *TNFSF4*), provides an important costimulatory signal supporting Th1 and Th2 responses, promoting expansion and survival of effector T cells and the generation of T memory cells. Although less understood, OX40/OX40L interactions also play a role in the activity and homeostasis of T regulatory cells. Signaling of this pair is tightly controlled, as OX40 is not expressed in resting T cells, only appearing approximately one day following initial activation. Similarly, OX40L is found only at sites of inflammation, first on the surface of antigen presenting cells, but later on diverse cell types including mast cells, suggesting a role distinct from T cell priming or memory cell generation. OX40-OX40L interactions are known to be involved in autoimmune disease, (*e.g.* SLE) likely acting through a disruption of tolerance. OX40 signaling within responding T cells renders them resistant to Treg- mediated suppression, and acts within the Treg cells to inhibit suppressive functions. In addition, sustained inflammatory response may result from excessive OX40-OX40L signaling and consequent increased survival of effector T-cells (see [24], [25]).

Two other regions showed suggestive associations, including SNPs between *MIR-552* and *GJB5* on Chromosome 1p34.3 (rs10915020 MAF = 0.84, P = 5.40×10^−07^, OR = 1.32), and near *ZNF365* on chromosome 10q21.2 (rs10995245 MAF = 0.35, P = 3.24×10^−07^ OR = 1.20). *ZNF365* is highly expressed in the brain and has been implicated in susceptibility to breast cancer, Crohn's Disease, and more recently, atopic dermatitis [26]–[28]. None of these reached genome-wide significance levels after correcting with the EMMAX [29] procedure in the current study, although nearly reaching or surpassing Bonferroni significance (P = 4.5×10^−07^)(Table S1). Increased sample size and replication will be needed to confirm these loci.

Our study, analyzing 1886 narcolepsy-cataplexy cases of European ancestry, is the largest collaborative cohort study of narcolepsy to date, including samples from across the United States, Canada and Europe, and representing the majority of available case samples of European ancestry. To preserve the statistical power afforded by this sample size, we elected not to split our cases into discovery and replication cohorts, and thus our study is limited by the lack of replication in an ethnically similar population. We identified two novel narcolepsy susceptibility genes, *CTSH* and *TNFSF4 (OX40L)*, and confirmed strong associations with HLA and TRA@. The two new loci identified outline with striking clarity that the key pathology underlying narcolepsy likely resides in the interaction between T cells and antigen presenting cells.

Although a role of antigen presentation to CD4 T cells is likely the primary susceptibility pathway for the disorder, narcolepsy was not associated with all components of this pathway as represented on the array. For example, we found no association at the p<10^−4^ threshold with the class II invariant chain, AEP and cathepsin B (*CTSB*) genes or, more surprisingly, with genes encoding other co-stimulatory molecules such as CD28, cytotoxic T-lymphocyte antigen-4 (*CTLA4*) and their cognate ligands, CD80 and CD86 (these have been involved in many other autoimmune disorders) (see Table S1). The present results also show limited overlap in susceptibility loci between narcolepsy and loci associated with classical autoimmune disorders, a fact that may be unsurprising based on the lack of readily identifiable autoantibodies, or other clear signs of inflammatory damage in the disease. To date, the TCR locus has only been observed in narcolepsy. Notably, we found no associations with loci widely shared among other autoimmune diseases such as interleukin genes and receptors (*IL2, IL21, IL12, IL2RA, IL23R*) acting in differentiation; *PTPN2* and 22, *SH2B3* and *TAGAP* involved in immune-cell activation and signaling; and *IRF5*, *TNFAIP3* involved in TNF signaling and innate immunity (Table S1). Together with findings implicating pandemic H1N1 influenza as a trigger, narcolepsy may offer a unique opportunity, furthering our understanding of how HLA class II presentation of foreign and self-antigens predispose to autoimmunity.

## Methods

### Ethics statement

Informed consent in accordance with governing institutions was obtained from all subjects. The research protocol at Stanford was approved by the IRB Panel on Medical Human Subjects.

### Samples

Cases included in this study all met criteria for narcolepsy/hypocretin deficiency (clear-cut cataplexy and DQB1*06:02 positive, or low cerebrospinal fluid hypocretin-1). Samples included 1301 patients sourced from the Stanford Center for Narcolepsy database (North America, and worldwide collaborators), and 585 samples contributed by the European narcolepsy network (EU-NN). ImmunoChip typing was performed at centers in the US and in Germany. Informed consent in accordance with governing institutions was obtained. Countries of origin included: United States (657), France (296), Italy (157), Germany (157), the Netherlands (111), Czech Republic (104), Canada (101), Austria (83), Denmark (74), Spain (51) and Norway (32). A further 63 cases came from Argentina, Australia, Finland, Israel, Poland, Portugal, Slovakia, Switzerland and Turkey, each with fewer than 20 samples. Control genotypes were contributed through multiple immunochip consortium collaborators including 4289 samples from the United Kingdom 1958 Birth Cohort, 3609 samples from selected European countries including Italy (1251), Netherlands (1173), Poland (529) and Spain (656), 980 samples from the German KORA cohort; 794 Samples from Cincinnati through CCHMC [30]; and 749 French samples (2 collaborators).

### Data analysis and statistics

Genotyping of cases was performed following Illumina's recommendation at U Virginia, USA, U of Munich, Germany, and Stanford University, Palo Alto, CA USA. NCBI build 36 (hg18) mapping was used as reference. Illumina manifest file Immuno_BeadChip_1149691_B.bpm was used in the majority of cases. In cases where file Immuno_BeadChip_11419691_A was used, map positions were converted to be consistent with 1149691_B, or omitted from the analysis. Genotypes were called using Illumina GeneExpress (Illumina GenomeStudio GenTrain2.0 algorithm), with extensive additional curation. Individuals with call rate under 98% (123 controls, 147 cases), and samples which were related (pi hat>0.2) were excluded from further analysis. Data from all sources were merged in forward-strand format. We identified 142,054 high quality SNPs with call rate above 99% (in both cases and controls separately), and passing HWE filtering in controls (P>1×10^−5^) using the Plink suite of software [31]. We excluded a broad region around the HLA complex (7,893 markers at Chr 6:24,067–35,474 kb) due to the strong LD effects with DQB1*06:02. This region contained nearly 3000 SNPs associated with narcolepsy at GWA significant levels. We additionally excluded SNPs with minor allele frequency below 1% (22,921 SNPs). Finally 111,240 high quality SNPs of MAF≥0.01 (including 91,804 MAF≥0.05) were selected for the analysis presented here. Principal components analysis (PCA) was performed to identify 162 outliers (133 controls, 29 cases; Golden Helix SVS, v7), and those were removed. Genome wide association analysis was performed using a variance component model implemented in EMMAX [29]. The EMMAX software does not return odds ratio or adjusted allele frequency data after correction for stratification. We therefore calculated OR and MAF (using Plink) for our tables based on a more homogeneous subsample of 8474 cases/controls based on principal components (EV 11.21<0.004, EV 4.12<0.01, see Figure S1). Linkage disequilibrium (as r^2^) values and haplotype analysis were calculated using Plink and Haploview [32] using data from our sample and/or from the 1000 genomes dataset [33]. QQ plots were generated using estlambda (http://www.genabel.org/GenABEL/estlambda.html), and Manhattan and PCA plots were made using SVS software.

### Imputation

Imputation and phasing of ImmunoChip genotypes were performed using Beagle v3.3 [34] against 4 European populations (286 individuals from CEU, TSI, GBR, IBS) in the 1000 genomes integrated data set (phase 1 release v3) within a 1 Mb window of the top hit at the CTSH and TNFSF4 loci. SNPs with an imputation R2 value≥0.8 (representing reliability of imputation) were considered in the analysis. Pairwise LD was calculated in Plink. Association P values in Figure 2 were calculated with Plink, as EMMAX would be inappropriate in this context, and therefore P values are slightly different than those presented in Table 2.

http://faculty.washington.edu/browning/beagle/beagle.html

http://bochet.gcc.biostat.washington.edu/beagle/1000_Genomes.phase1_release_v3/

### Cleavage prediction

Sequence used: MWATLPLLCAGAWLL[G/R]VPVCGAAELCVNSLEKFHFKSWTSKHRKTYSTEEYHHRLQTFAS

SignalIP: http://www.cbs.dtu.dk/services/SignalP/ Both alleles are predicted to have normal cleavage

SigPred: http://bmbpcu36.leeds.ac.uk/prot_analysis/Signal.html Predicts cleavage unlikely for Arg variant.

## Supporting Information

Figure S1 Principal components analysis of the study population. Eigenvectors 1 versus 2 in cases and controls are displayed (a–c). Dashed lines in panel a indicate boundaries of a subset of 8474 samples used to calculate OR and allele frequencies (see Methods).
(TIF)

Table S1 Top ranking non-HLA narcolepsy risk variant signals to P = 1×10^−4^. All non-HLA variants (MAF>1% passing QC measures), and with P values<1×10^−4^. are displayed. Chr.: Chromosome; BP: position according to NCBI build 36 (Hg18) coordinates; MAF_N: minor allele frequency in narcolepsy (_N) and controls (_C); P: P value according to variance component model (EMMAX). EMMAX does not provide OR (Odds Ratio) or adjusted allele frequencies, therefore MAF, OR, and 95% confidence intervals (CI) were calculated with Plink on subset of 8474 samples with the greatest homogeneity (see Figure S1 ; EV 11.21<0.004, EV 4.12<0.01). Threshold for significance using Bonferroni correction: 4.5×10^−7^.
(DOCX)
