# Supplementary figures and images for: ImmunoChip Study Implicates Antigen Presentation to T Cells in Narcolepsy
Source: PLoS Genet. 2013 Feb 14;9(2):e1003270. doi: 10.1371/journal.pgen.1003270 (PMC3573113; doi:10.1371/journal.pgen.1003270)

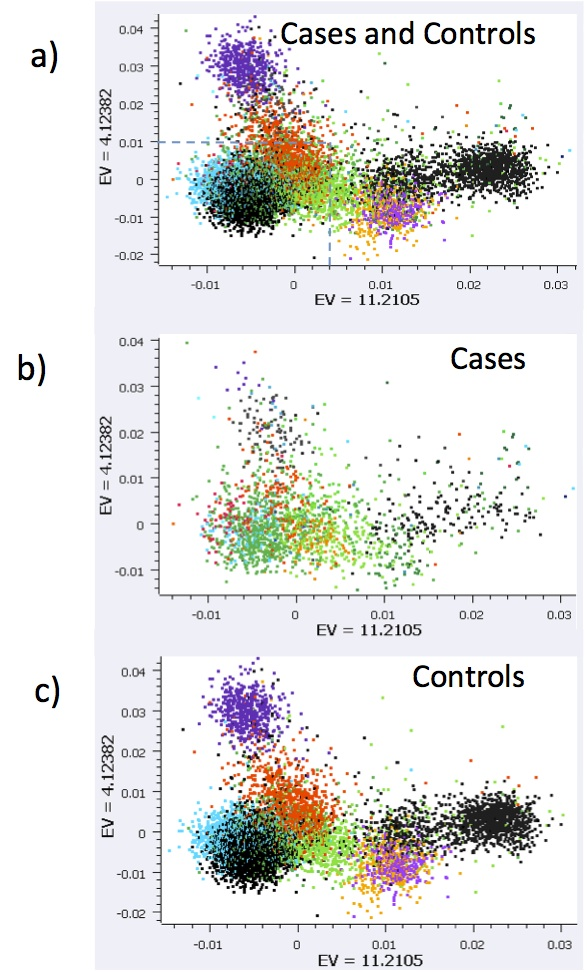

Supplement: Figure S1 — Principal components analysis of the study population. Eigenvectors 1 versus 2 in cases and controls are displayed (a–c). Dashed lines in panel a indicate boundaries of a subset of 8474 samples used to calculate OR and allele frequencies (see Methods). (TIF) [file pgen.1003270.s001.tif]
