# Supplementary material for: ImmunoChip Study Implicates Antigen Presentation to T Cells in Narcolepsy
Source: PLoS Genet. 2013 Feb 14;9(2):e1003270. doi: 10.1371/journal.pgen.1003270 (PMC3573113; doi:10.1371/journal.pgen.1003270)
Supplement: Table S1 — Top ranking non-HLA narcolepsy risk variant signals to P = 1×10^−4^. All non-HLA variants (MAF>1% passing QC measures), and with P values<1×10^−4^. are displayed. Chr.: Chromosome; BP: position according to NCBI build 36 (Hg18) coordinates; MAF_N: minor allele frequency in narcolepsy (_N) and controls (_C); P: P value according to variance component model (EMMAX). EMMAX does not provide OR (Odds Ratio) or adjusted allele frequencies, therefore MAF, OR, and 95% confidence intervals (CI) were calculated with Plink on subset of 8474 samples with the greatest homogeneity (see Figure S1 ; EV 11.21<0.004, EV 4.12<0.01). Threshold for significance using Bonferroni correction: 4.5×10^−7^. (DOCX) [file pgen.1003270.s002.docx]

Supplementary Table 1 Top ranking non-HLA narcolepsy risk variant signals to P=1 x10^-4^

| SNP | Chr. | Bp | MAF_N | MAF_C | P | OR | CI | Localization | Risk allele |
| --- | --- | --- | --- | --- | --- | --- | --- | --- | --- |
| rs1154155 | 14 | 22072524 | 0.229 | 0.148 | 8.87 x10^-30^ | 1.72 | 1.54-1.91 | TCRA J region | G |
| rs34593439 | 15 | 77022012 | 0.136 | 0.105 | 1.78 x10^-08^ | 1.34 | 1.18-1.52 | CTSH | A |
| rs34843303 | 15 | 77021525 | 0.143 | 0.111 | 2.79 x10^-08^ | 1.35 | 1.19-1.53 | CTSH | C |
| rs7553711 | 1 | 171398531 | 0.346 | 0.285 | 4.08 x10^-08^ | 1.33 | 1.21-1.46 | TNFSF4 | C |
| rs3861950 | 1 | 171422915 | 0.372 | 0.311 | 5.42 x10^-08^ | 1.31 | 1.20-1.43 | TNFSF4 | C |
| rs4081545 | 1 | 171426139 | 0.371 | 0.311 | 7.30 x10^-08^ | 1.31 | 1.20-1.43 | TNFSF4 | C |
| rs6665456 | 1 | 171407388 | 0.345 | 0.285 | 7.40 x10^-08^ | 1.32 | 1.21-1.45 | TNFSF4 | C |
| rs4090391 | 1 | 171413713 | 0.345 | 0.285 | 7.73 x10^-08^ | 1.32 | 1.21-1.45 | TNFSF4 | T |
| rs6676785 | 1 | 171411640 | 0.345 | 0.285 | 8.08 x10^-08^ | 1.32 | 1.21-1.45 | TNFSF4 | G |
| rs10127495 | 1 | 171415294 | 0.345 | 0.285 | 8.49 x10^-08^ | 1.32 | 1.21-1.45 | TNFSF4 | C |
| rs10157163 | 1 | 171399588 | 0.345 | 0.285 | 9.67 x10^-08^ | 1.32 | 1.21-1.45 | TNFSF4 | T |
| rs6691738 | 1 | 171418659 | 0.345 | 0.285 | 9.88 x10^-08^ | 1.32 | 1.20-1.45 | TNFSF4 | G |
| rs4916312 | 1 | 171412980 | 0.345 | 0.285 | 9.90 x10^-08^ | 1.32 | 1.21-1.45 | TNFSF4 | A |
| rs4916313 | 1 | 171431410 | 0.370 | 0.310 | 1.57 x10^-07^ | 1.30 | 1.19-1.43 | TNFSF4 | C |
| rs7154229 | 1 | 171420927 | 0.368 | 0.310 | 2.24 x10^-07^ | 1.30 | 1.19-1.42 | TNFSF4 | T |
| rs10995245 | 10 | 64061381 | 0.390 | 0.348 | 3.24 x10^-07^ | 1.20 | 1.09-1.31 | ZNF365 | A |
| rs10915020 | 1 | 34930303 | 0.108 | 0.084 | 5.40 x10^-07^ | 1.32 | 1.15-1.52 | MIR552 to GJB5 | A |
| rs16836898 | 1 | 34932196 | 0.108 | 0.084 | 7.28 x10^-07^ | 1.32 | 1.14-1.52 | MIR552 to GJB5 | T |
| rs1932768 | 1 | 34938559 | 0.107 | 0.084 | 1.36 x10^-06^ | 1.31 | 1.14-1.51 | MIR552 to GJB5 | T |
| rs306336 | 1 | 88745467 | 0.037 | 0.023 | 1.94 x10^-06^ | 1.65 | 1.30-2.09 | RP11-76N22.3 to PKN2 | C |
| rs3784539 | 15 | 77006243 | 0.166 | 0.136 | 3.09 x10^-06^ | 1.27 | 1.13-1.43 | CTSH | T |
| rs12148472 | 15 | 77018533 | 0.159 | 0.130 | 3.88 x10^-06^ | 1.26 | 1.12-1.42 | CTSH | C |
| rs16845506 | 1 | 171408423 | 0.258 | 0.214 | 4.90 x10^-06^ | 1.28 | 1.16-1.42 | TNFSF4 | G |
| rs2834168 | 21 | 33572661 | 0.286 | 0.313 | 5.30 x10^-06^ | 0.88 | 0.80-0.96 | IL10RB | G |
| rs10127727 | 1 | 171417587 | 0.258 | 0.214 | 5.61 x10^-06^ | 1.28 | 1.16-1.41 | TNFSF4 | A |
| rs10465507 | 1 | 171429062 | 0.279 | 0.235 | 6.33 x10^-06^ | 1.26 | 1.15-1.39 | TNFSF4 | A |
| rs10912561 | 1 | 171431188 | 0.279 | 0.235 | 6.57 x10^-06^ | 1.26 | 1.15-1.39 | TNFSF4 | T |
| rs725790 | 1 | 34921035 | 0.087 | 0.071 | 6.93 x10^-06^ | 1.26 | 1.08-1.47 | Mir552 to GJB5 | G |
| rs10798264 | 1 | 171429980 | 0.279 | 0.235 | 7.26 x10^-06^ | 1.26 | 1.14-1.39 | TNFSF4 | A |
| rs1027327 | 3 | 8581839 | 0.126 | 0.095 | 8.68 x10^-06^ | 1.36 | 1.19-1.56 | LMCD1\Dyxin | C |
| rs12409611 | 1 | 88746060 | 0.037 | 0.024 | 1.02 x10^-05^ | 1.58 | 1.25-2.00 | RP11-76N22.3 to PKN2 | C |
| rs7525284 | 1 | 171435020 | 0.316 | 0.266 | 1.19 x10^-05^ | 1.27 | 1.16-1.39 | TNFSF4 | A |
| rs6671025 | 1 | 88752035 | 0.037 | 0.024 | 1.22 x10^-05^ | 1.58 | 1.24-2.00 | RP11-76N22.3 to PKN2 | A |
| rs7075349 | 10 | 64097655 | 0.365 | 0.332 | 1.28 x10^-05^ | 1.16 | 1.06-1.27 | ZNF365 | G |
| rs10912560 | 1 | 171431080 | 0.283 | 0.239 | 1.39 x10^-05^ | 1.26 | 1.14-1.38 | TNFSF4 | G |
| rs11160606 | 14 | 100371014 | 0.201 | 0.169 | 1.60 x10^-05^ | 1.24 | 1.11-1.38 | MEG3 | T |
| rs13343108 | 1 | 171434853 | 0.282 | 0.239 | 1.71 x10^-05^ | 1.25 | 1.13-1.38 | TNFSF4 | C |
| rs11811856 | 1 | 171438296 | 0.316 | 0.269 | 2.87 x10^-05^ | 1.26 | 1.15-1.38 | TNFSF4 | G |
| rs11811788 | 1 | 171417350 | 0.291 | 0.242 | 3.00 x10^-05^ | 1.29 | 1.17-1.42 | TNFSF4 | G |
| rs12098699 | 10 | 64066796 | 0.228 | 0.201 | 3.63 x10^-05^ | 1.17 | 1.05-1.30 | ZNF365 | G |
| rs10761653 | 10 | 64074040 | 0.251 | 0.289 | 3.86 x10^-05^ | 0.82 | 0.75-0.91 | ZNF365 | T |
| rs17817382 | 17 | 50417179 | 0.106 | 0.137 | 3.86 x10^-05^ | 0.75 | 0.65-0.86 | STXBP4 | G |
| rs16988402 | 22 | 28943841 | 0.035 | 0.020 | 4.85 x10^-05^ | 1.74 | 1.36-2.23 | HORMAD2 to LIF | T |
| rs67087719 | 22 | 28945511 | 0.035 | 0.020 | 4.85 x10^-05^ | 1.74 | 1.36-2.23 | HORMAD2 to LIF | T |
| rs4745874 | 10 | 64083815 | 0.250 | 0.289 | 4.93 x10^-05^ | 0.82 | 0.75-0.91 | ZNF365 | T |
| rs8039661 | 15 | 77046064 | 0.385 | 0.423 | 5.31 x10^-05^ | 0.86 | 0.78-0.94 | CTSH region | T |
| rs1363761 | 16 | 26434772 | 0.342 | 0.315 | 5.41 x10^-05^ | 1.13 | 1.03-1.24 | Gene desert | C |
| rs11679585 | 2 | 40544972 | 0.215 | 0.186 | 5.73 x10^-05^ | 1.19 | 1.07-1.33 | SLC8A1 | A |
| rs4237305 | 10 | 64077857 | 0.228 | 0.265 | 5.78 x10^-05^ | 0.83 | 0.74-0.91 | ZNF365 | T |
| rs10761654 | 10 | 64075652 | 0.229 | 0.265 | 6.90 x10^-05^ | 0.82 | 0.74-0.91 | ZNF365 | A |
| rs6479830 | 10 | 64088745 | 0.251 | 0.288 | 7.28 x10^-05^ | 0.83 | 0.75-0.92 | ZNF365 | T |
| rs58340896 | 2 | 102000000 | 0.012 | 0.023 | 7.74 x10^-05^ | 0.53 | 0.36-0.77 | IL1R1 | C |
| rs12890215 | 14 | 100000000 | 0.255 | 0.223 | 7.84 x10^-05^ | 1.19 | 1.08-1.32 | MEG3 | C |
| rs479454 | 6 | 117000000 | 0.426 | 0.397 | 7.85 x10^-05^ | 1.13 | 1.03-1.23 | DSE | T |
| rs2393907 | 10 | 64077365 | 0.229 | 0.265 | 8.54 x10^-05^ | 0.83 | 0.75-0.91 | ZNF365 | C |
| rs57623551 | 15 | 77048132 | 0.430 | 0.392 | 8.64 x10^-05^ | 1.17 | 1.07-1.28 | CTSH region | G |
| rs2044264 | 1 | 88738386 | 0.026 | 0.017 | 9.70 x10^-05^ | 1.54 | 1.17-2.04 | RP11-76N22.3 to PKN2 | C |

All non-HLA variants ((MAF >1% passing QC measures), and with P values < 1 x10^-4.^ are displayed. Chr.: Chromosome; BP: position according to NCBI build 36 (Hg18) coordinates; MAF_N: minor allele frequency in narcolepsy (_N) and controls (_C); P: Pvalue according to variance component model (EMMAX). EMMAX does not provide OR (Odds Ratio) or adjusted allele frequencies, therefore MAF, OR, and 95% confidence intervals (CI) were calculated with Plink on subset of 8474 samples with the greatest homogeneity (see Suppl Figure 2; EV 11.21 < 0.004, EV 4.12 < 0.01). Threshold for significance using Bonferroni correction: 4.5x10^-7^.
